# Clinical validation of smartphone-based activity tracking in peripheral artery disease patients

**DOI:** 10.1038/s41746-018-0073-x

**Published:** 2018-12-11

**Authors:** Raheel Ata, Neil Gandhi, Hannah Rasmussen, Osama El-Gabalawy, Santiago Gutierrez, Alizeh Ahmad, Siddharth Suresh, Roshini Ravi, Kara Rothenberg, Oliver Aalami

**Affiliations:** 1 Division of Vascular & Endovascular Surgery, Department of Surgery, Stanford University, Stanford, CA 94305 USA; 2 Division of Vascular Surgery, Veterans Affairs Palo Alto Health Care System, Palo Alto, CA 94304 USA; 3 Precision Health and Integrated Diagnostics Center at Stanford, Stanford University, Stanford, CA 94305 USA

**Keywords:** Peripheral vascular disease, Diagnostic markers, Peripheral vascular disease, Diagnostic markers

## Abstract

Peripheral artery disease (PAD) is a vascular disease that leads to reduced blood flow to the limbs, often causing claudication symptoms that impair patients’ ability to walk. The distance walked during a 6-min walk test (6MWT) correlates well with patient claudication symptoms, so we developed the VascTrac iPhone app as a platform for monitoring PAD using a digital 6MWT. In this study, we evaluate the accuracy of the built-in iPhone distance and step-counting algorithms during 6MWTs. One hundred and fourteen (114) participants with PAD performed a supervised 6MWT using the VascTrac app while simultaneously wearing an ActiGraph GT9X Activity Monitor. Steps and distance-walked during the 6MWT were manually measured and used to assess the bias in the iPhone CMPedometer algorithms. The iPhone CMPedometer step algorithm underestimated steps with a bias of −7.2% ± 13.8% (mean ± SD) and had a mean percent difference with the Actigraph (Actigraph-iPhone) of 5.7% ± 20.5%. The iPhone CMPedometer distance algorithm overestimated distance with a bias of 43% ± 42% due to overestimation in stride length. Our correction factor improved distance estimation to 8% ± 32%. The Ankle-Brachial Index (ABI) correlated poorly with steps (*R* = 0.365) and distance (*R* = 0.413). Thus, in PAD patients, the iPhone’s built-in distance algorithm is unable to accurately measure distance, suggesting that custom algorithms are necessary for using iPhones as a platform for monitoring distance walked in PAD patients. Although the iPhone accurately measured steps, more research is necessary to establish step counting as a clinically meaningful metric for PAD.

## Introduction

Peripheral arterial disease (PAD) affects over 10 million people in the United States.^1,2^ While intermittent claudication (IC) is the classic early symptom for PAD, many patients are asymptomatic or have exertional leg symptoms other than IC.^3^ Medical management with smoking cessation, exercise, aspirin, and statin therapy are the first line of therapy, however, surgical interventions are employed to improve PAD patient’s walking ability when there is a “disabling” loss of mobility.^4^

The current standard of care for diagnosis and post-operative surveillance of PAD consists of ankle-brachial indices (ABIs) and/or arterial duplex scans, both conducted in the clinic. Given the association between severity of walking disability and arterial disease burden,^5^ patient-reported claudication symptoms (i.e., leg cramping) are often monitored during clinic visits as well. However, arterial duplex scans and ABI results do not always correlate with symptoms, and self-reported patient data can be unreliable.

Initially developed as a fitness test for the Air Force,^6^ the 6-min walk test (6MWT) is an objective tool that is commonly used in clinic to assess functional capacity in chronic obstructive pulmonary disease and congestive heart failure (CHF).^7,8^ The primary measure of a 6MWT is the 6-min walk distance (6MWD), the distance walked in 6-min on a linear 100-ft course. Though the 6MWT is not traditionally used in the PAD space, it has been shown to correlate with claudication symptoms^9–12^ and functional capacity in patients with PAD.^3,10,12–14^ Despite the 6MWT’s simplicity, it is typically administered by trained personnel in a clinical setting.

Smartphones have accelerometers and gyroscopes that can measure physical activity and have been shown to be effective tools for collecting clinical data at high resolution and on a large scale.^15–17^ Brooks et al.^18^ demonstrated that smartphone apps can effectively and reliably administer a 6MWT both in clinic and at home for patients with CHF or pulmonary hypertension, suggesting that smartphones may be a promising platform for remotely monitoring functional capacity. We hypothesize that a remotely-administered 6MWT could more accurately reflect the day-to-day function of PAD patients and provide a more patient-centric metric for patients’ functional limitations. Interestingly, while studies have assessed the use of Fitbits and the ActiGraph pedometer to remotely monitor “free-living physical activity”,^19^ to the authors’ knowledge no study to date has assessed the validity of a smartphone-based 6MWT in the PAD population. With over 75% of the general population, and, notably, 46% of participants aged 65+ reporting smartphone ownership,^20^ we believe that a smartphone-based 6MWT could change the paradigm for PAD surveillance by allowing physicians to track functional limitations in patients with PAD and to measure patient responses to intervention longitudinally.

To explore the use of a smartphone-based monitoring tool in the PAD population, we created an iPhone app that administers a 6MWT as well as PAD-specific survey questionnaires. In this study we aim to assess the feasibility of our 6MWT app, “VascTrac,” to serve as a platform for performing 6MWTs in patients with PAD by (1) evaluating the accuracy of the iPhone’s step and distance tracking algorithms in the PAD population, and (2) assessing the concordance of the iPhone algorithms with the ActiGraph GT9X.

## Results

### Study sample

**Fig. 1:**
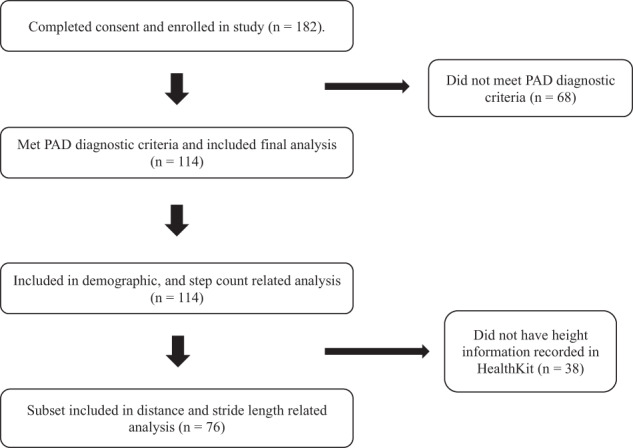
Patient recruitment flowchart

One hundred and fourteen individuals who met diagnostic criteria for PAD (see Methods) were included in the final analysis (Fig. 1). An overview of patient characteristics is provided in Table 1. A majority of the study population was male (77%). Mean age of the population was 69.5 years, mean height was 1.72 m, and mean BMI was 26.9 kg/m^2^. Eighty percent of participants were current or former smokers. Comorbidities included hypertension (70.1%), diabetes (33.3%), and coronary artery disease (41.2%). 22.8% of patients used a cane or walker as a walking aid.

**Table 1:** Participant characteristics

Characteristic	Total (*N* = 114)
Age, mean (SD)	69.5 (13.1) years
Gender, *n* (%)	
Male	88 (77.2%)
Female	26 (22.8%)
Height, mean (SD)	1.72 (0.1) meters
Weight, mean (SD)	79.6 (16.3) kg
BMI, mean (SD)	26.9 (4.7) kg/m^2^
BMI, *n* (%)	
Underweight, <18.5 kg/m^2^	4 (3.5%)
Healthy weight, 18.5–24.9 kg/m^2^	37 (32.5%)
Overweight, 25.0–29.9 kg/m^2^	47 (41.2%)
Obese, ≥30.0 kg/m^2^	26 (22.8%)
Smoking status, *n* (%)	
Current smoker	19 (17.0%)
Former smoker	71 (63.4%)
Never smoked	22 (19.6%)
Comorbidities, *n* (%)	
Hypertension	80 (70.1%)
Diabetes	38 (33.3%)
Coronary artery disease	47 (41.2%)
PAD diagnosis method, *n* (%)	
ABI < = 0.9	71 (62.3%)
TBI < = 0.7	13 (11.4%)
History of PAD Surgery	30 (26.3%)
Walking aid, *n* (%)	
Cane	12 (10.5%)
Walker	14 (12.3%)
None	88 (77.2%)

*SD* standard deviation, *BMI* body mass index, *PAD* peripheral artery disease, *ABI* arterial brachial index, *TBI* toe brachial index

### iPhone step and distance algorithms

**Fig. 2:**
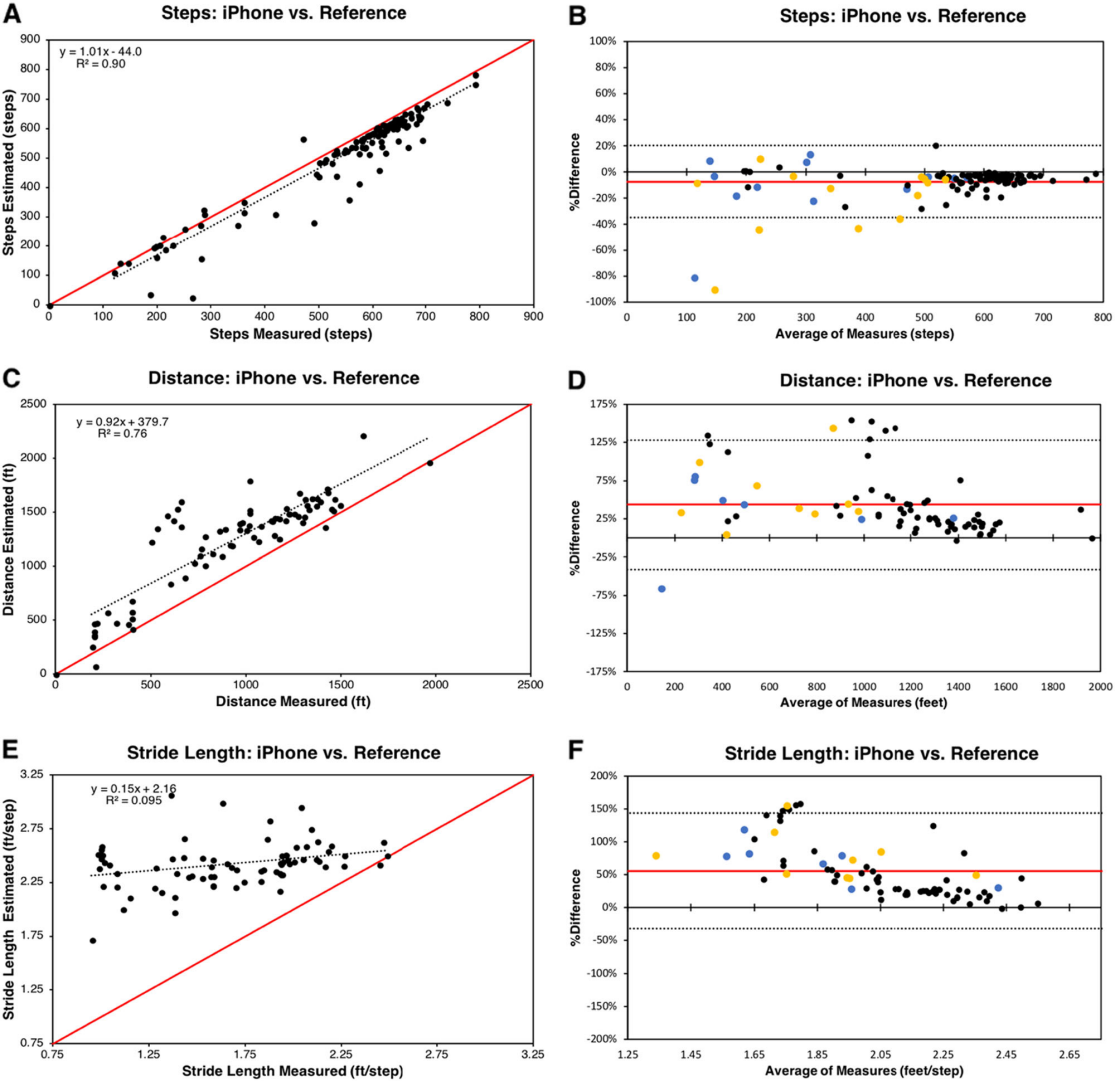
iPhone accuracy analysis. Device accuracy was assessed using scatter plots and Bland-Altman plots. **a** Scatter plot of iPhone versus manual step counting. **b** Bland-Altman plot of iPhone versus manual step counting. **c** Scatter plot of iPhone distance versus measured distance. **d** Bland-Altman plot of iPhone distance versus observed distance. **e** Scatter plot of iPhone average stride length versus average measured stride length. **f** Bland-Altman plot of iPhone average stride length versus average measured stride length. In scatter plots, black dots indicate participants, solid red lines demarcate y = x line, and black dashed lines represent regression lines. The regression equation and coefficient of determination are depicted in the graph’s top left corner. In Bland-Altman plots, solid red lines demarcate the bias and dashed black lines indicate the 95% limits of agreement. Black data points = no walking aid. Yellow data points = walkers. Blue data points = canes

We evaluated the accuracy of the iPhone CMPedometer step counting and distance estimation algorithms by comparing each to their respective reference standards (manual step counting and track distance). We found that the iPhone CMPedometer step counting algorithm underestimated steps during the 6MWT, with a bias of −7.2% ± 13.8% (mean ± standard deviation) (Fig. 2a, b). By contrast, the distance algorithm overestimated the 6MWD, with a bias of 43% ± 42% (Fig. 2c, d). BA plots revealed no systematic differences or trends in the error.

### Stride length

Previous research has shown that PAD can cause patients’ stride length to decrease,^21^ so we hypothesized that the CMPedometer distance algorithm’s overestimation of 6MWD was due to faulty calculation of stride length in PAD patients. We therefore assessed the accuracy of each user’s estimated average stride length against the reference standards (Fig. 2e, f). We found that the CMPedometer algorithms overestimated the average stride length of study participants, with a bias of 56% ± 44%. Comparison of stride length measured by the CMPedometer distance algorithm and the reference standard using a BA plot demonstrated that error decreased as stride length increased; specifically, when a participant’s average stride length was less than 2 feet per step by reference standard, the error in the estimated average stride length increased (Fig. 2f). Linear regression analysis of the relationship between stride length and distance measured by the CMPedometer distance algorithm revealed a correlation of −0.746 (*P* < 0.001, CI: −0.843, −0.556) (Table 2). Given the systematic overestimation of distance we were able to able to reduce distance bias to 8% ± 32% by applying a linear correction factor of 0.75 (based on the average overestimation of the CMPedometer distance algorithm (33%)) to each participant’s CMPedometer-calculated distance.

**Table 2:** Pearson coefficients for linear regressions

	ActiGraph steps % error			CMPedometer step algorithm % error			CMPedometer distance algorithm % error		
Predictor covariate	*R*	*P*-value	95% CI^a^	*R*	*P*-value	95% CI	*R*	*P*-value	95% CI
Distance	0.230	0.017	[0.0424, 0.403]	0.244	0.009	[0.062, 0.411]	−0.557	< 0.001	[−0.695, −0.379]
Average stride length	0.273	0.004	[0.088, 0.440]	0.238	0.011	[0.055, 0.405]	−0.746	< 0.001	[−0.832, −0.636]
Steps	0.154	0.114	[−0.037, 0.334]	0.184	0.051	[0.001, 0.357]	−0.216	0.061	[−0.421, 0.010]
Age	−0.121	0.217	[−0.305, 0.072]	−0.164	0.084	[−0.339, 0.022]	0.006	0.959	[−0.221, 0.233]
Weight (kg)	0.013	0.894	[−0.177, 0.202]	0.115	0.226	[−0.071, 0.293]	0.062	0.597	[−0.166, 0.283]
Height (m)	0.139	0.153	[−0.052, 0.321]	0.060	0.527	[−0.126, 0.242]	0.139	0.230	[−0.089, 0.354]
BMI	−0.059	0.550	[−0.246, 0.133]	0.106	0.263	[−0.080, 0.285]	−0.005	0.965	[−0.230, 0.226]
Sex	−0.009	0.925	[−0.299, 0.181]	0.038	0.690	[−0.148, 0.221]	−0.067	0.563	[−0.288, 0.161]
Walking Aid	−0.050	0.607	[−0.238, 0.141]	−0.175	0.064	[−0.349, 0.010]	0.125	0.283	[−0.104, 0.341]
BMI^a^	−0.018	0.853	[−0.207, 0.172]	0.119	0.208	[−0.067, 0.298]	0.012	0.919	[−0.214, 0.237]

Linear regressions were calculated for continuous and categorical covariates to identify the relationship between covariates and device percent error. The Pearson coefficient (*R*) is reported for each regression. For error in iPhone CMPedometer distance estimation, average stride length was found to have the highest correlation (*R* = −0.746), indicating that the iPhone distance estimation error increases as participant stride length decreases

*CI* confidence interval, *BMI* body mass index

^a^The second use of BMI is as a categorical variable

### ActiGraph accuracy

**Fig. 3:**
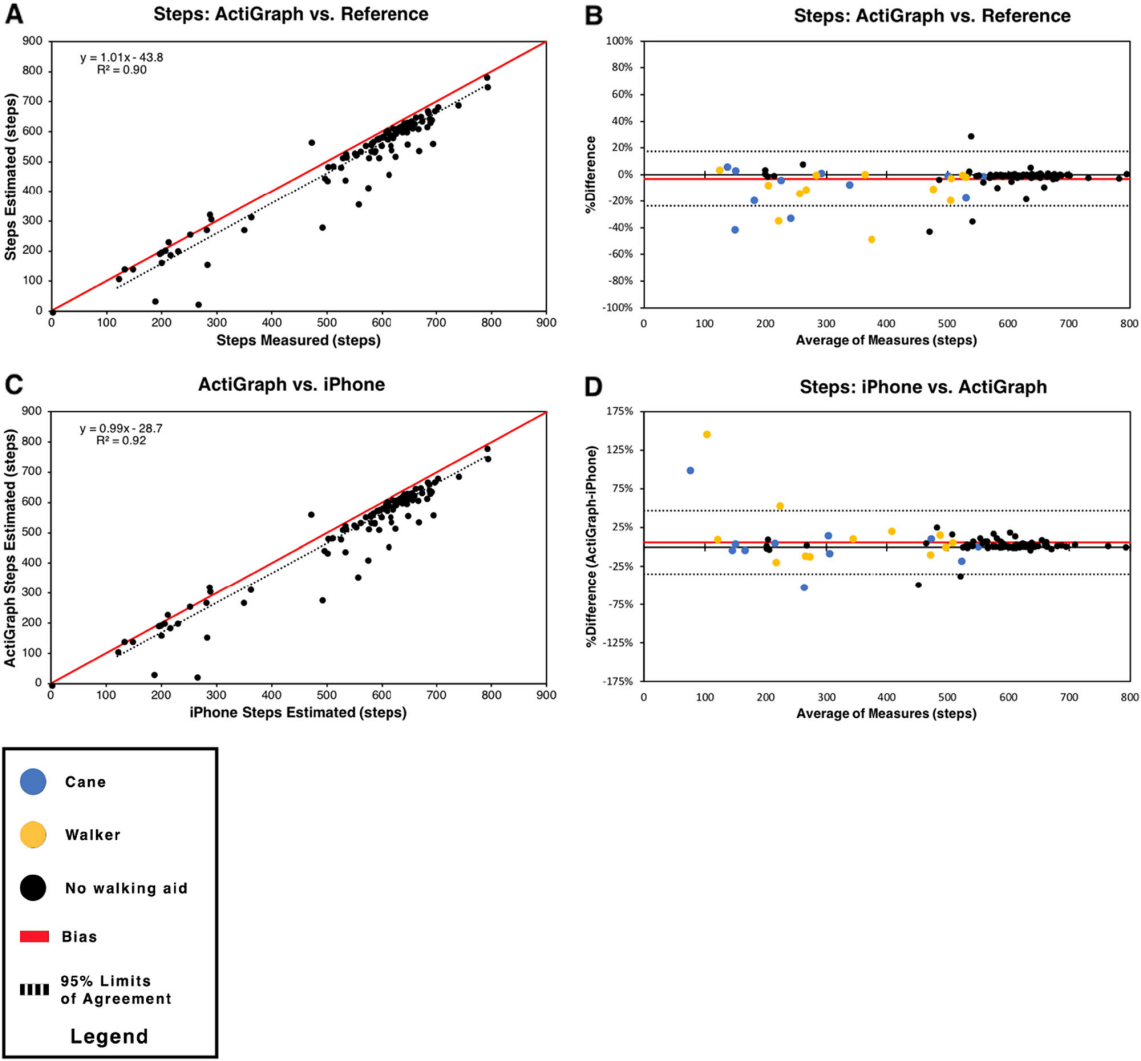
ActiGraph accuracy analysis. Device accuracy was assessed using scatter plots and Bland-Altman plots. **a** Scatter plot of ActiGraph versus manual step counting. **b** Bland-Altman plot of ActiGraph versus manual step counting. **c** Scatter plot of ActiGraph and iPhone step estimation. **d** Bland-Altman plot comparing iPhone steps and ActiGraph steps. In scatter plots, black dots indicate participants, solid red lines demarcate y = x line, and black dashed lines represent regression lines. The regression equation and coefficient of determination are depicted in the graph’s top left corner. In Bland-Altman plots, solid red lines demarcate the bias and dashed black lines indicate the 95% limits of agreement. Black data points = no walking aid. Yellow data points = walkers. Blue data points = canes

In order to determine how the iPhone step counting algorithm compared with a research-grade pedometer, we also assessed the ActiGraph’s step counting error by comparing steps counted by the ActiGraph to the reference standard (manual step counting). Similar to the CMPedometer step counting algorithm, the ActiGraph step counting algorithm underestimated steps during the 6MWT with a bias of −3.1% ± 10.3% (Fig. 3a, b). When the ActiGraph and iPhone were assessed for their step counting concordance, they were found to have a strong correlation *R* = 0.96 and a mean difference (ActiGraph-iPhone) of 5.7% ± 20.5% (Fig. 3c, d). BA plots revealed no systematic differences or trends in the error.

### Error analysis

A variety of covariates were analyzed as predictors of accuracy using linear regression (Table 2). For both the iPhone CMPedometer step algorithm and the ActiGraph, linear regression analysis did not reveal any strong predictors for device percent error in step counting. Based on the Pearson correlation coefficient, all covariates were weak predictors and explained a very small percentage of variability in the device percent error. Some covariates were statistically significant predictors of step counting error, however they had small correlation coefficients. For error in iPhone CMPedometer distance estimation, average stride length was found to be have the highest correlation (*R* = −0.764), indicating that the iPhone distance estimation error increases as participant stride length decreases.

### ABI sub-analysis

**Fig. 4:**
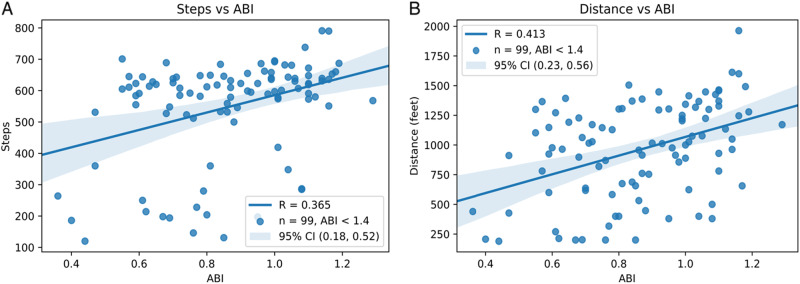
Steps vs Ankle-brachial index and distance vs. ankle-brachial index. Ankle-brachial indices (ABI) were measured for each leg, and the lower ABI of the two legs were used for each participant. Participants with ABI’s > 1.4 or who required toe-brachial indices were excluded (*n* = 15). **a** Steps are plotted as measured by the reference, manual step counting. **b** Distance is plotted as measured by the reference, distance traveled in the 6-min walk test

Because the ABI is one of the key diagnostic tests for PAD, we were interested in assessing the potential correlation between physical activity metrics and ABI value. This was accomplished by comparing patients’ reference standard measurements (i.e., manual step count or track distance) to their ABI scores. Patients with ABI > 1.4 (considered a non-diagnostic reading), as well as patients who required a TBI, were excluded from this analysis (*n* = 15). Comparison revealed that the correlation was weak between ABI and steps (*R* = 0.365) as well as ABI and distance (*R* = 0.413) (Fig. 4a, b).

## Discussion

Previous studies have assessed the CMPedometer algorithms’ accuracy in small samples of healthy individuals,^22–24^ but few have assessed their accuracy in diseased populations. In this study, we have demonstrated that the iPhone’s CMPedometer algorithm grossly overestimates distance in PAD patients performing a 6MWT due its overestimation of user stride length. As a result, in their current state, the built-in CMPedemoter APIs cannot be used in apps like VascTrac for clinical measures where accurate distance estimation is necessary. Brooks et al.^18^ processed raw accelerometer data from the iPhone to build custom algorithms for distance estimation in the CHF population for assessing a user’s 6MWD. Although they evaluated the iPhone 4s, which does not have built-in pedometer features such as the CMPedometer algorithm, their methodology may be necessary to build clinically useful iPhone applications that leverage distance metrics. To correct for the systematic overestimation of stride length, we attempted to implement a simple correction factor that others could use to improve distance accuracy. However, the correction factor was obtained through a retrospective analysis and will require further testing in a prospective study setup. It should be acknowledged that the Apple Watch attempts to mitigate the limitations in stride length estimation by using GPS to calibrate distance estimation over time. Although we did not evaluate the Apple Watch in our study, it may be an important tool for future studies.

At the same time, our results demonstrate that the iPhone has strong concordance with the ActiGraph for step counting and that both devices accurately measure steps compared to the reference standard in the PAD population. While the 6MWD serves as an important marker for multiple diseases beyond PAD, such as pulmonary artery hypertension, CHF, and chronic obstructive pulmonary disease,^10,25,26^ step counting has also shown strong associations with a number of health variables across multiple cross-sectional studies.^27^ Thus, using step counts may be an alternative path forward for future activity-related studies that leverage the iPhone APIs. However, further validation will be necessary to use step counts for monitoring severity of PAD.

McDermott et al.^5^ have shown that the ABI is significantly associated with walking distance among men and women with PAD. Although these studies report p-values that may be statistically significant, our data suggest that these associations are weak trends with weak correlations. If one of the major indications to treat PAD is significant functional limitation, yet the ABIs we measure poorly correlate with distance, it is worth investigating whether the ABI is the correct tool for monitoring severity of disease in these patients. The 6MWT has been shown to effectively measure response to PAD therapy, and performance on the 6MWT is not only indicative of a patient’s disease, but also predicts mortality in patients with PAD.^28–30^ We propose transitioning to more patient-centric activity-based outcomes for patients with PAD, such as 6MWT performance, and using activity monitors with accelerometers to develop even better functional metrics that could be used both diagnostically and for postoperative surveillance.

Furthermore, we are entering an exciting time where novel consumer devices like iPhones and Fitbits are infiltrating many areas of clinical medicine. These devices enable data collection on an unprecedented scale and provide APIs to extract metrics and build platforms for monitoring disease. However, APIs also operate as a “black box,” obscuring the user from its algorithms and accuracy. Recently, the FDA has started to develop new programs, such as the Digital Health Software Precertification Program, for regulating software as a medical device (SaMD). This program approves company software development processes rather than the software updates themselves. While these programs would enable faster approval of SaMD, they will also empower companies to maintain the proprietary nature of their algorithms. This would allow a single iteration of an algorithm to immediately obsolesce years of previous research and require additional validation for every clinical indication, which is not feasible for the scientific community. We therefore welcome a regulatory approval process that requires transparency in the algorithm iteration processes to ensure accuracy and quality with each update. Ideally, in the future, software will be open source with detailed documentation for others to validate. Such transparency would enable the research community to more easily study these algorithms, tailor them to various disease populations, and adapt in the event of algorithm updates.

The authors acknowledge that there were a number of limitations to our study that should be noted when considering our results. For example, a disproportionate percentage of study participants were male (77%). This was due to the primarily male demographic at the Palo Alto Veterans Affairs Medical Center, one of our two study enrollment sites, and this gender skewing may limit the generalizability of our conclusions. A second limitation is the fact that the iPhone was held in the participant’s hand during the 6MWT, while the ActiGraph was placed on their hip. Ideally, the location of the ActiGraph would have been matched to the same hand that held the phone in order to minimize positioning differences. However, co-localizing devices was challenging because the devices were set up prior to each study visit. Since the location of the ActiGraph must be included during its setup process, it was difficult to anticipate which hand a participant would hold the phone in—especially for those who used walking aids. Several studies have shown agreement between the ActiGraph on the wrist and the hip, which should help increase the validity of our results.^31^ Additionally, we recognize that on a day-to-day basis, PAD patients will not carry their phone in their hand at all times. However, for the purposes of this study, we intended to evaluate the accuracy of the CMPedometer algorithms during a 6MWT, where a participant will usually hold the phone in their hand while walking. In a follow-up study, we will evaluate the feasibility of measuring step counts in real world settings to remotely monitoring PAD beyond the 6MWT. A third limitation is that we did not compare other smartphone or smartwatch devices such as Android phones or Fitbits at this time because of the heterogeneity of accelerometer hardware. Consequently, some of this study’s results may not be generalizable to other activity monitoring devices.

In this study, we demonstrated that the iPhone CMPedometer distance estimation algorithm has poor accuracy in patients with PAD, likely due to lack of correction for an individual’s stride length. Additionally, we showed that the iPhone CMPedometer step counting algorithm is highly concordant with the ActiGraph and reference standard. We therefore feel confident that steps measured by an iPhone app can be used as a metric for remote physical activity tracking in PAD patients. Moving forward, we plan to assess repeatability and reliability of an unsupervised 6MWT in the PAD population, while simultaneously collecting passive data on daily step count and distance walked. These future studies will work toward the ultimate goal of developing a validated functional capacity tool for remote surveillance pre-medical and post-medical or surgical intervention.

## Methods

### VascTrac app development

As an initial step, we set out to determine the most accurate position for a patient to hold the smartphone in order to collect activity metrics during a 6MWT. Using nine healthy participants, we measured the error in step counting and distance measurement for a number of phone positions: including phone in the hand, pocket, and in a purse or bag (Supplementary Table 1). The step and distance measurement algorithms were provided by Apple’s built in CoreMotion (CM) PedometerData Application Programming Interface (API) with functions ‘numberOfSteps’ and ‘distance’. After determining that holding the phone in the hand is the most accurate location, we then built a native iOS app, called VascTrac, utilizing Apple’s ResearchKit (http://researchkit.org) framework, with functionality to: (1) consent and enroll patients; (2) survey patients to obtain pertinent medical and surgical history; (3) administer a 6MWT according to the American Thoracic Society (ATS) 6MWT Guidelines; (4) record step counts, estimated distance walked using Apple’s CMPedometerData API and (5) wirelessly transmit this data to a HIPAA-compliant centralized database.

Medical history surveys recorded medications, comorbidities, and recent ABI or toe-brachial index (TBI) reading. Surgical history surveys collected previous vascular procedures and date(s) of procedure(s). iPhones SE, 6, 7, and 7 Plus running iOS 10 (Apple Inc., Cupertino, Ca.) were used. Similarity in data collection across different iPhone models was previously established by our team via testing on an athletic track. Data was transferred and stored in real time to HIPAA-compliant Microsoft Azure servers (Microsoft Corp., Redmond, Wa.). The code and data that support the findings of this study are available from the corresponding author upon reasonable request. It should be noted that even though every step of the onboarding was designed to be user-centric, such that a study participant could enroll and complete the study independently, a researcher walked every patient through the app for this “supervised” study.

### Participant recruitment and characterization

Patients who presented to the Stanford Hospital vascular clinic or Palo Alto Veterans Affairs Hospital vascular clinic for evaluation of a PAD diagnosis, or for follow-up after a previous diagnosis of PAD, were approached for recruitment into this study between June 2017 and September 2017. Reasons for PAD diagnostic evaluation included patient reports of claudication-like symptoms, absent pedal pulses noted by primary care physician, or being at increased risk for PAD based on comorbidities. For inclusion in the study, participants were required to be: (1) English-speaking, (2) ≥18 years old, (3) willing to share medical/surgical history, and (4) willing to perform a supervised 6MWT at the end of their appointment. Exclusion criteria were developed using the ATS 6MWT Guidelines and included: (1) being wheelchair bound, (2) being immobile, (3) experiencing chest pain in the last 30 days, or (4) exhibiting lower extremity open wounds.^21^

If deemed eligible and willing to take part in the study, participants were given a study device and directed to a consent process within the VascTrac app. Participants who completed the consent step were considered enrolled into the study (*n* = 182). During analysis, participants were classified as part of the PAD cohort if they had an ABI score ≤ 0.9, a TBI score ≤ 0.7, or a previous surgical intervention for PAD (*n* = 114). Medical, surgical, and ABI histories were obtained verbally from participants, recorded in the VascTrac app, and verified by chart review. Within the PAD cohort, only participants who had height information correctly entered into HealthKit were included in the distance and stride length analysis (*n* = 76). Participants who did not meet criteria for PAD categorization were excluded from central analysis (*n* = 68). Figure 1 describes the flow of participants recruited and ultimately included in final analysis.

To reduce sampling bias, study devices were given to each eligible patient for the research study and returned at the end of the clinic visit. Each patient’s height and weight were entered into Apple HealthKit (https://developer.apple.com/healthkit/) from within the VascTrac app.

### Ethics approval

The study was approved by the Stanford Institutional Review Board (IRB) and all participants provided informed consent. All relevant ethical regulations were complied with and the study was registered at ClinicalTrials.gov (NCT03048890).

### Supervised 6MWT

**Fig. 5:**
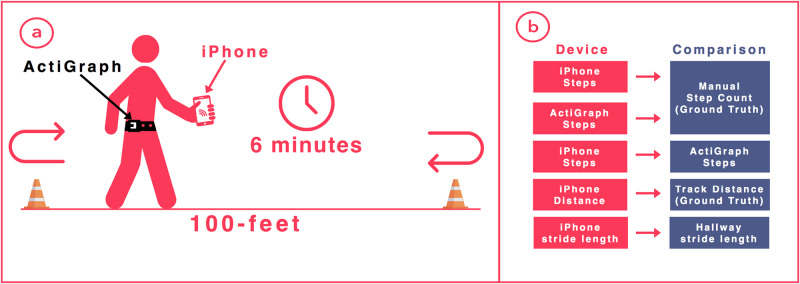
6MWT study design and metric comparison paradigm. **a** Schematic illustrating the 6MWT study design. Patients walked back and forth along a 100-foot course for 6 min or until they could walk no further, while an ActiGraph, iPhone, and human observed and measured walking metrics (e.g., steps, distance). **b** Schematic illustrating the paradigm used for device analysis

Consenting patients performed a 6MWT along a pre-measured 100-foot course with an iPhone (Apple Inc., Cupertino, Ca) in one hand and an ActiGraph GT9X (ActiGraph Corp., Pensacola, FL) attached at the waistband on the right hip. Because the ActiGraph location must be included as an initialization parameter during the device’s setup, the right hip was chosen as a standardized location to minimize error in the device initialization process. Use of walking aids, such as canes or walkers, was noted. Because patients with walkers required two-hands on a walker, the iPhone was placed in a front shirt or pant pocket for these individuals. One coordinator manually counted steps, which were compared to the VascTrac app and the ActiGraph GT9X. Manual step counting methods were validated separately (Supplementary Table 2). A second coordinator calculated distance using total laps completed on the 6MWT course, which was used as a reference standard for comparison to distance measured by the VascTrac app. This coordinator also documented changes in gait, symptoms, and whether patients stopped the 6MWT early. The manual step count and distance walked served as the reference standards to which we compared results from the smartphone and ActiGraph. A depiction of our study design can be found in Fig. 5.

### Manual step counting validation

To establish our reference standard for steps, one of the trained clinical coordinators manually counted steps for each participant. To establish each coordinator’s step counting error, we performed five videotaped trials in the same hallway under the same walk test conditions. During each trial, the coordinators manually counted number of steps taken by the test subject. The videos were then analyzed in slow motion by two different raters in order to determine the true number of steps taken in each trial. Using the videotaped trial steps as the ground truth, we were able to quantify each coordinator’s accuracy. In order to participate in manual step counting during the study, coordinators were required to have a mean percent error of one percent or less.

### ActiGraph parameters processing

The ActiGraph GT9X (ActiGraph Corp., Pensacola, Fl.) was used to capture data using its 3-axis accelerometers. ActiGraph LLC’s proprietary ActiLife6 software was used to set up the device and download the data (Supplementary Table 3).

### Statistical analysis

Distribution of all measurements were assessed using box-and-whisker plot. Next, three main analyses were conducted: (1) iPhone CMPedometer distance estimation error, (2) iPhone CMPedometer step counting error, and (3) iPhone CMPedometer step counting concordance with ActiGraph GT9X step counting. For each analysis, scatter plots were created to visualize the correlation between algorithms and reference standard measurements, and Bland-Altman (BA) plots were established to evaluate the differences between the two measurement methods. The BA plot is a simple way to compare two quantitative methods and assess any bias between the two methods.^32^ The BA plot also estimates an agreement interval within which 95% of the differences between two measurement techniques fall. Typically, BA plots use the magnitude of the error on the *y*-axis, but in instances where the magnitude of the error varies across measurements, it has been established that the percent error can be used.^33^

To quantify the error in iPhone measured steps and distance compared to the reference standard, the percent error for each participant was calculated using the following formula:

$${\mathrm{Error}} = \left( {{\mathrm{Measurement}} - {\mathrm{reference}}} \right){\mathrm{/reference}} \ast 100\%$$

The average stride length for each participant was calculated by dividing 6MWD by total step count. A distance correction was calculated using a linear factor calculated by comparing the average stride length measured by the references to the average stride length recorded by the CMPedometer. Finally, linear regressions were performed to evaluate any associations with device error and the following covariates: distance, stride length, steps, age, weight, height, BMI, sex, walking aids, and categorical BMI classification. Categorical covariates were encoded into binary variables for regressions. Statistical analysis was performed using Python 2.7 (Anaconda, Inc., Austin, TX) and Microsoft Excel 2016 (Microsoft Corp., Redmond, Wa.).

### Code availability statement

All code that was written to process and analyze the data can be available upon reasonable request from the corresponding author (O.A.).

## Electronic supplementary material

Supplementary Tables and Figures

## Data Availability

The data that support the findings of the study are available upon reasonable request from the corresponding author (O.A.). The data is not publicly available due to containment of Personal Health Information (PHI).

## Electronic supplementary material

**Supplementary information** accompanies the paper on the *npj Digital Medicine* website (10.1038/s41746-018-0073-x).
