# Author Correction: Clinical validation of smartphone-based activity tracking in peripheral artery disease patients

**DOI:** 10.1038/s41746-020-00316-0

**Published:** 2020-08-07

**Authors:** Raheel Ata, Neil Gandhi, Hannah Rasmussen, Osama El-Gabalawy, Santiago Gutierrez, Alizeh Ahmad, Siddharth Suresh, Roshini Ravi, Kara Rothenberg, Oliver Aalami

**Affiliations:** 1 Division of Vascular & Endovascular Surgery, Department of Surgery, Stanford University, Stanford, CA 94305 USA; 2 Division of Vascular Surgery, Veterans Affairs Palo Alto Health Care System, Palo Alto, CA 94304 USA; 3 Precision Health and Integrated Diagnostics Center at Stanford, Stanford University, Stanford, CA 94305 USA

**Keywords:** Peripheral vascular disease, Diagnostic markers, Peripheral vascular disease, Diagnostic markers

Correction to: *npj Digital Medicine*10.1038/s41746-018-0073-x, published online 11 December 2018

The original version of the published Article had a mistake in the first two sentences of the Author contributions section. The Author contributions have been updated to the following: R.A., N.G., H.R., and O.E. should be considered as co-first authors.

The HTML and PDF versions of the Article have been corrected.

