# Supplementary material for: Clinical validation of smartphone-based activity tracking in peripheral artery disease patients
Source: NPJ Digit Med. 2018 Dec 11;1:66. doi: 10.1038/s41746-018-0073-x (PMC6550212; doi:10.1038/s41746-018-0073-x)
Supplement: Supplementary file 1 — Supplementary Tables and Figures [file 41746_2018_73_MOESM1_ESM.docx]

**SUPPLEMENTARY MATERIALS**

| **Trial Number** | **N** | **Phone Location** | **Distance (m)** | **Mean % Error: Steps** | **Mean % Error: Distance** |
| --- | --- | --- | --- | --- | --- |
| 1 | 9 | Fixed in hand | 100 | 4.7 | 9.5 |
| 2 | 9 | Swinging in hand | 100 | 3.7 | 0.3 |
| 3 | 9 | Front pocket | 100 | 12.8 | 15.0 |
| 4 | 9 | Back pocket | 100 | 15.6 | 16.4 |
| 5 | 9 | Purse/Bag | 100 | 2.6 | 9.3 |

**Supplementary Table 1**. Five trials were performed to assess the most accurate location of the iPhone for measuring steps and distance in nine health participants. Each participant test the location of the iPhone in five different positions: 1)iPhone fixed in the hand, minimizing any movement of the phone during the walk, 2)iPhone in hand in a natural swinging motion, 3)iPhone placed in a front pocket of pants or shorts, 4)iPhone in the back pocket of pants or shorts, and 5)iPhone placed in a purse or bag. Steps measured by the phone were compared to the reference, manually counted steps. Distance measured by the phone was compared to the reference, a 100-m pre-measured track.

|  | **Mean Steps Counted** | **Mean % Error** | **SD^a^ of % Error** |
| --- | --- | --- | --- |
| **Rater 1** | 592 | 0.03% | 0.18% |
| **Rater 2** | 591 | -0.04% | 0.25% |
| **Rater 3** | 592 | -0.08% | 0.34% |
| **Rater 4** | 591 | -0.16% | 0.41% |
| **Rater 5** | 591 | -0.21% | 0.42% |
| **Rater 6** | 590 | -0.31% | 0.61% |
| **Rater 7** | 589 | -0.49% | 0.61% |

**^a^SD: standard deviation**

**Supplementary Table 2: Rater Accuracy.** A separate validation study to evaluate the accuracy of the eight manual step counters involved in the study was performed. Each step counter, i.e., rater, counted steps in five videotaped trials and their average for the five trials was calculated. The percent error for each rater was then tabulated using the number of steps measured in the video as ground truth. The mean steps measured in the video was 593 steps.

| **Parameter** | Setting |
| --- | --- |
| **ActiLife version** | 6.13.3 |
| **Sample rate** | 100Hz |
| **Epoch** | 1 second |
| **Inertial measurement unit** | Enabled |
| **Low frequency extension** | Enabled |
| **Limb** | Waist |

**Supplementary Table 3: ActiGraph parameter settings.** After each walk test, raw accelerometer data was downloaded and processed using ActiLife software. Raw GT3X files were converted to AGD files with an Epoch of 1 second and low frequency extension (LFE) enabled. Wear time window for each patient was manually established, and the ActiLife scoring function was used to calculate steps.

**
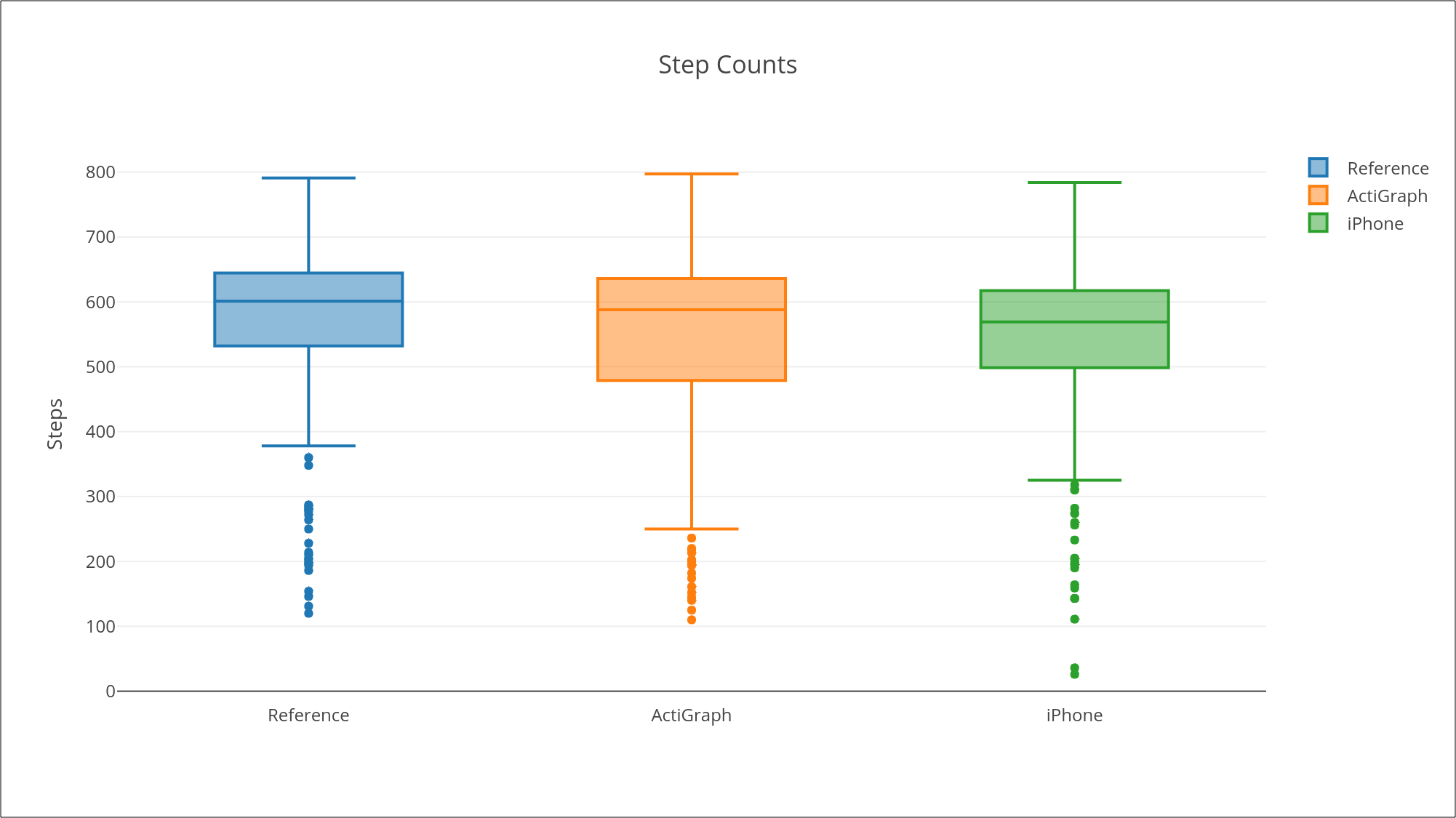
**

**
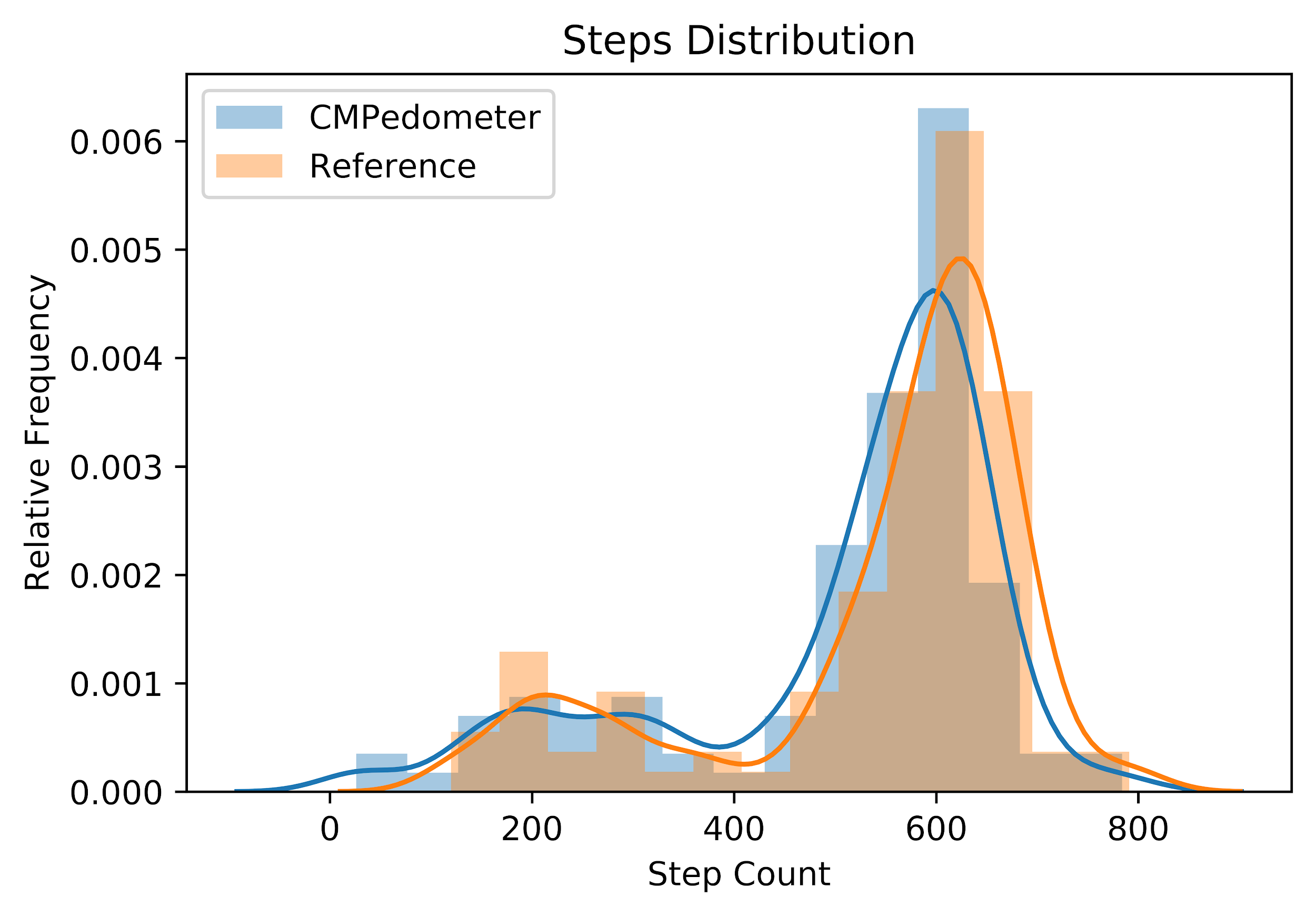

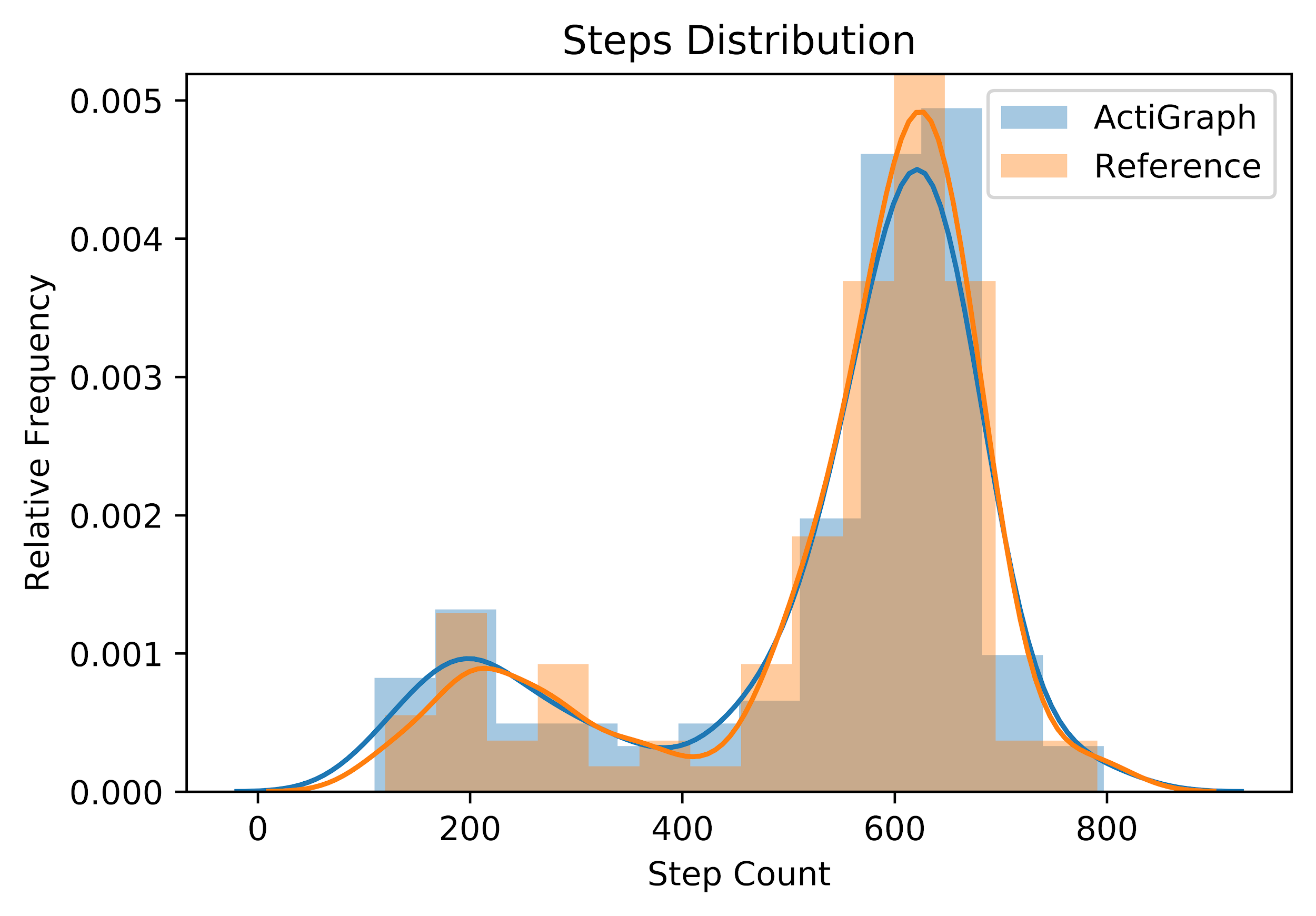
**

**Supplementary Figure 1: Step Count Box-and-whisker Plot.** All step count measurements collected in the study, grouped by device or reference (manually counted steps). The boxplots indicate the 25% quantile, median, and 75% quantiles of the step count measurements. Data points more than 1.5 IQR values above the 75% quantile are treated as outliers and indicated with a dot.

**Supplementary Figure 2: Distance Measurement Box-and-whisker Plot**

**
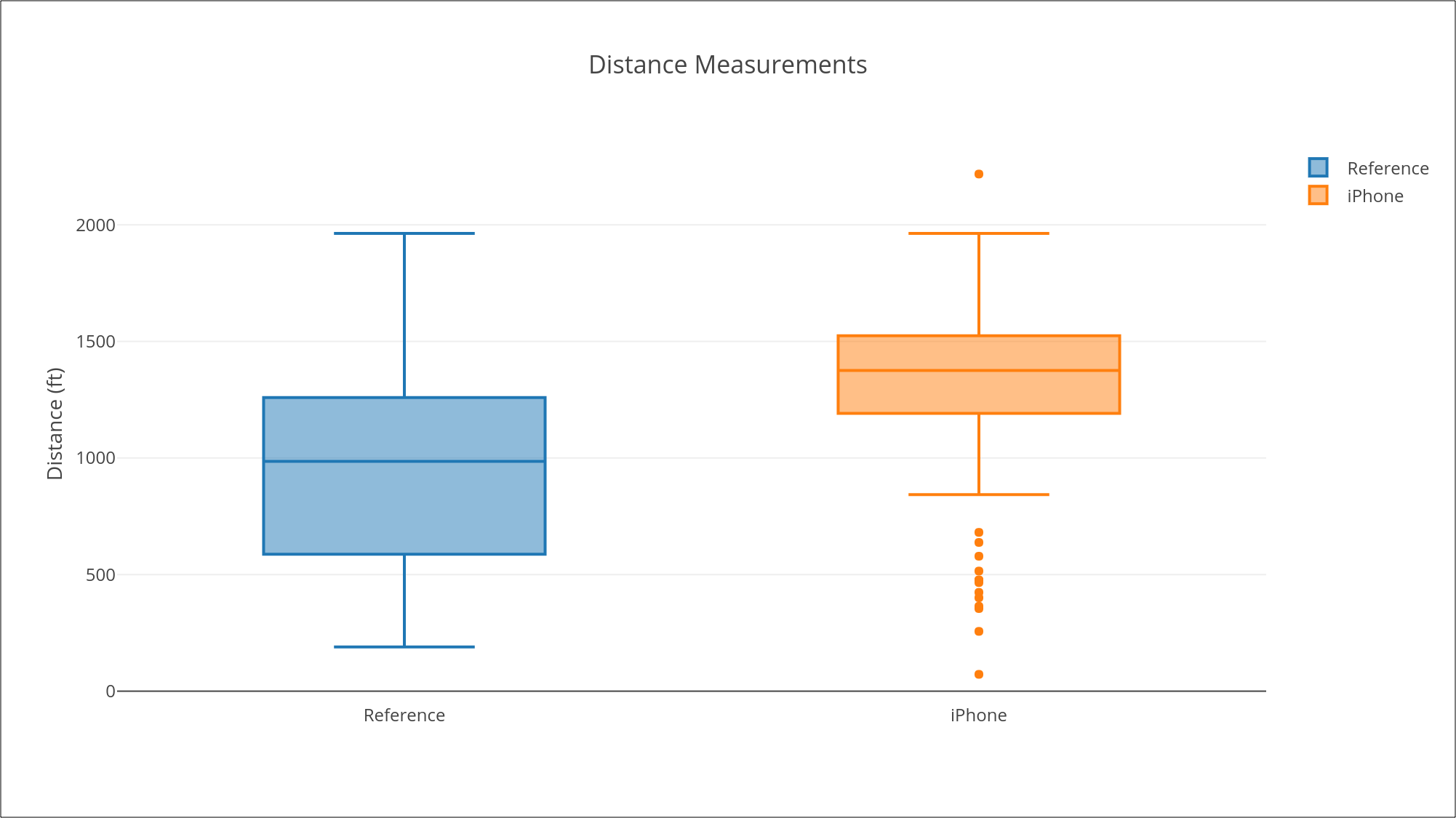
**

**
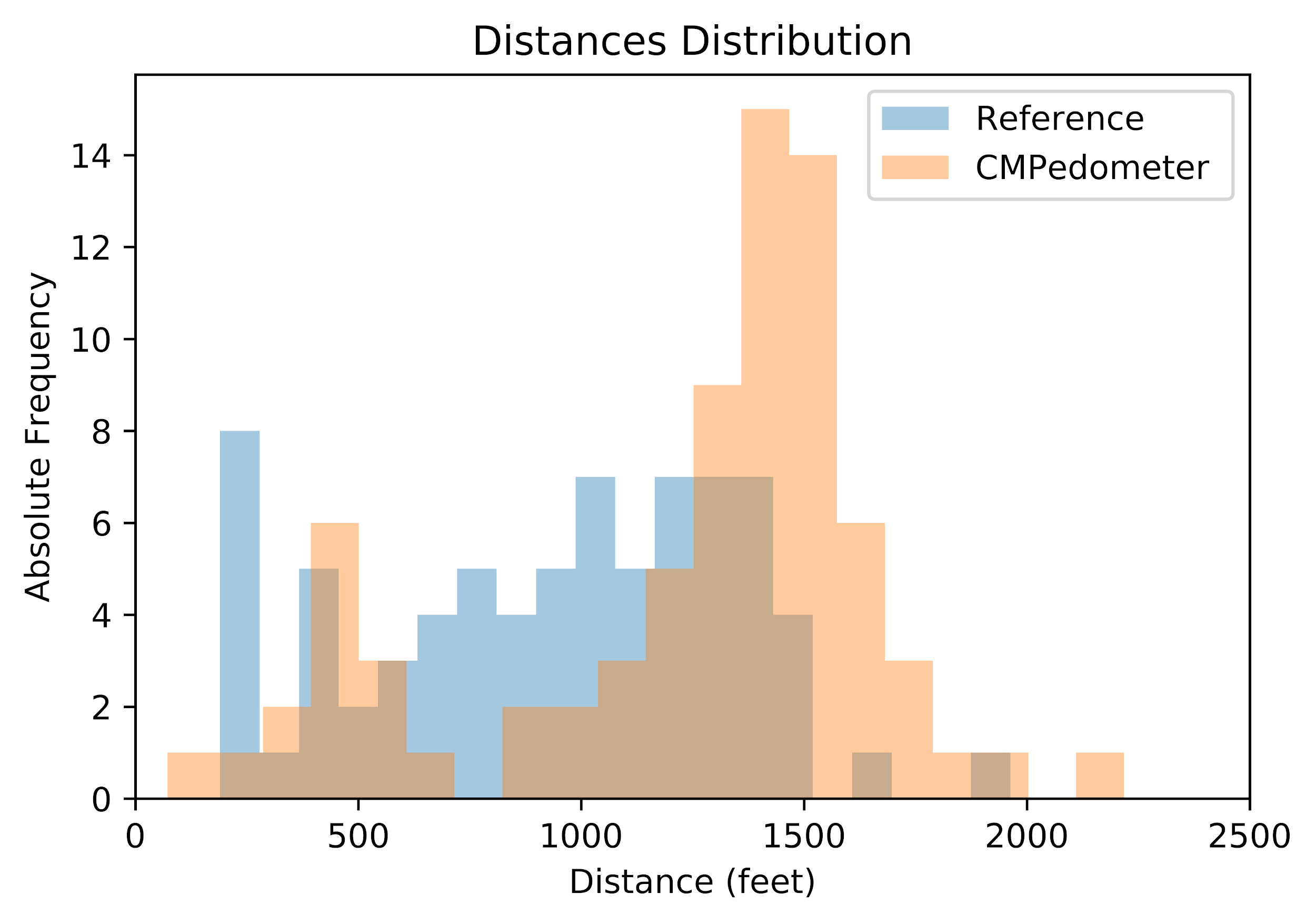

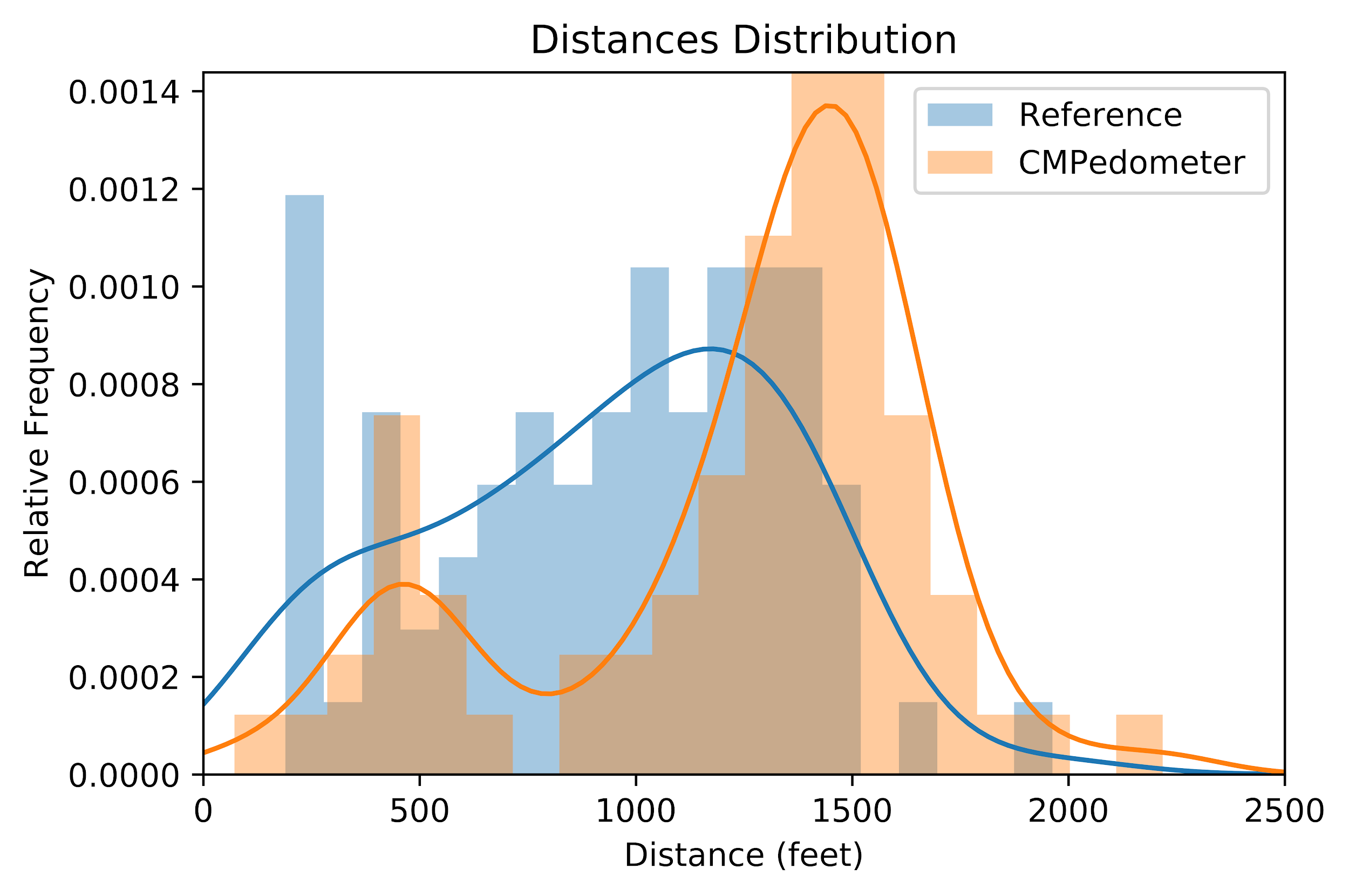
**

**Supplementary Figure 2: Distance Measurement Box-and-whisker Plot.** All distance measurements collected in the study for the iPhone and reference (manually measured distance). The boxplots indicate the 25% quantile, median, and 75% quantiles of the step count measurements. Data points more than 1.5 IQR values above the 75% quantile are treated as outliers and indicated with a dot.

**
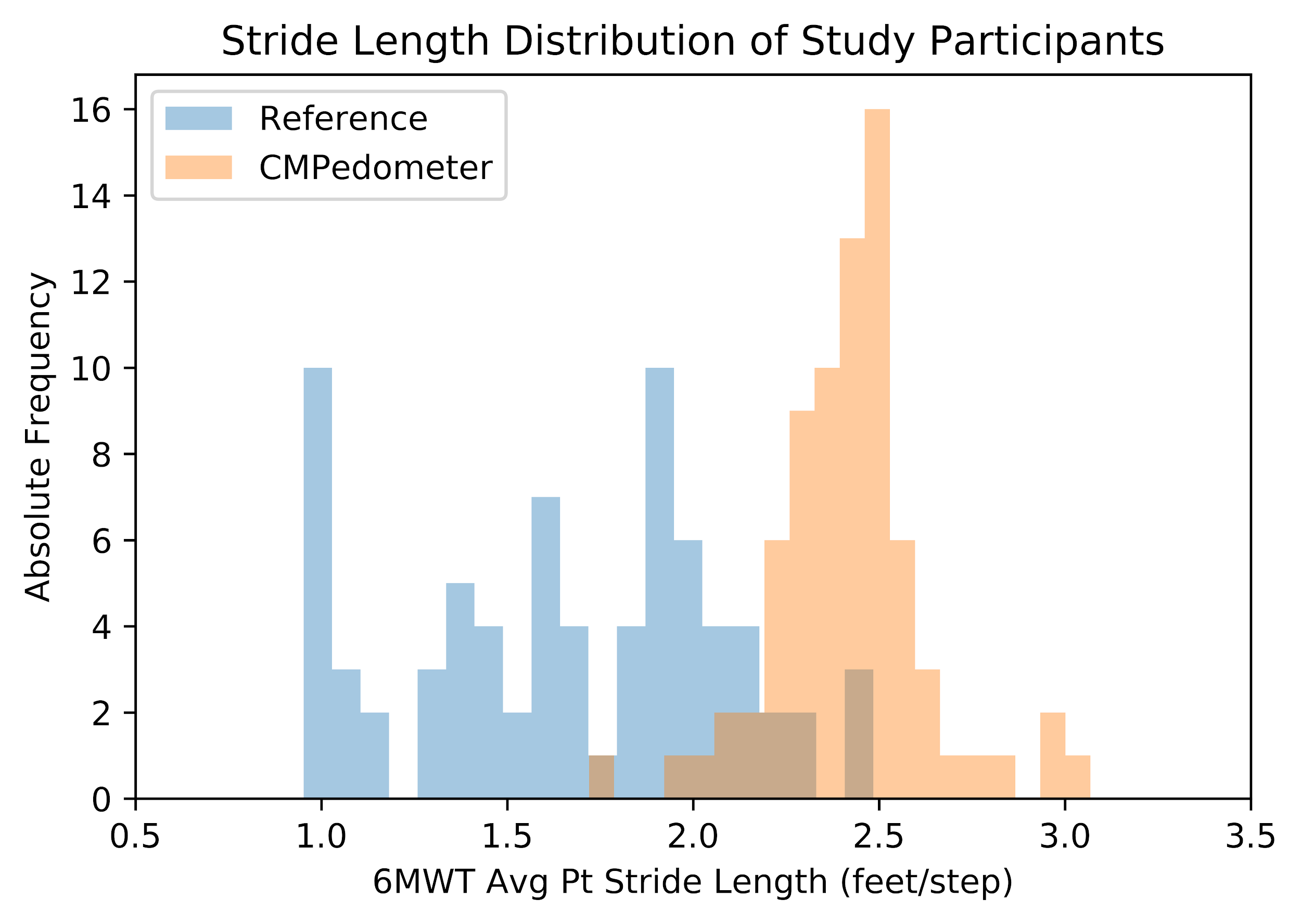

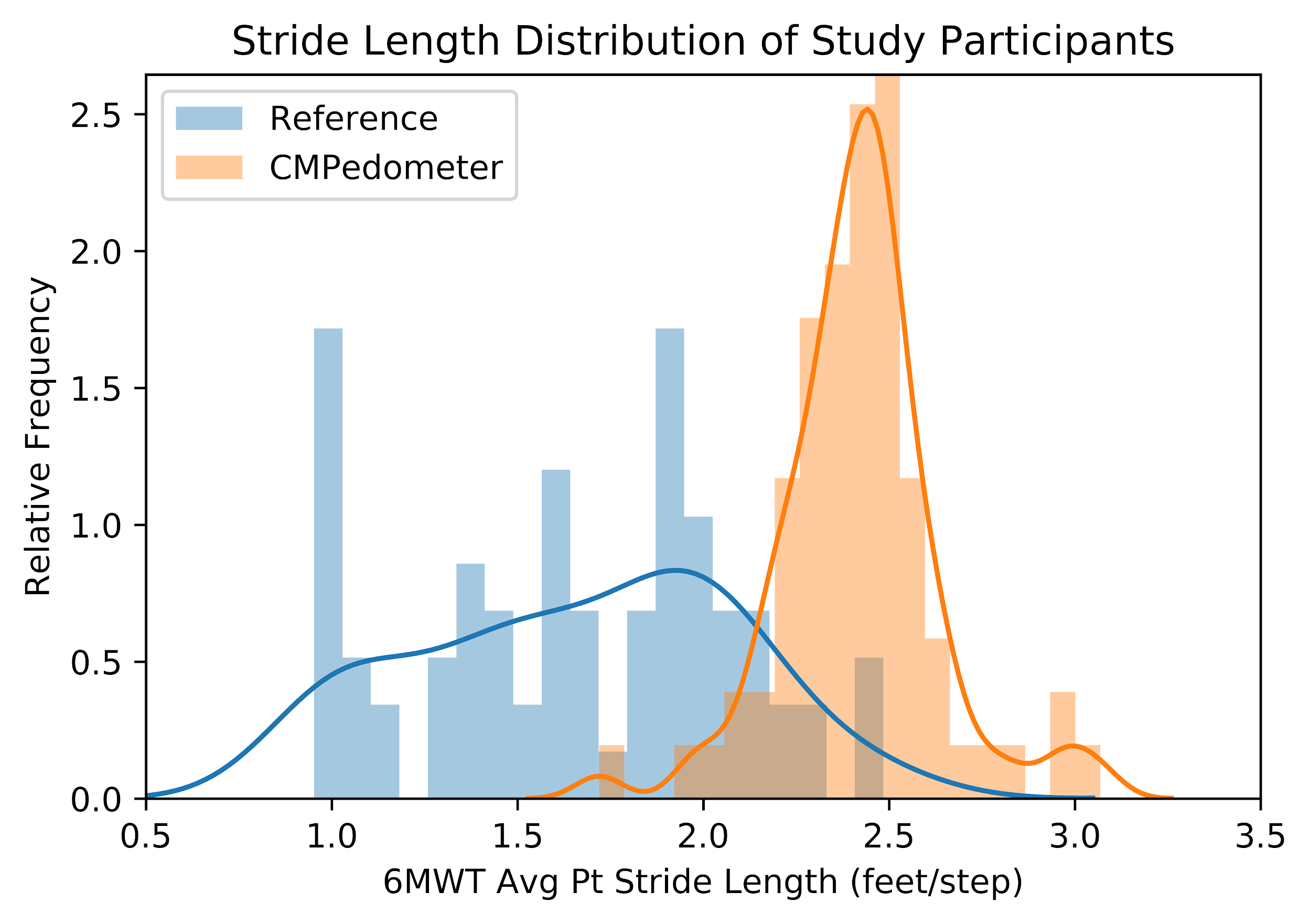
**

**Supplementary Figure 3: Stride Length Participant Distribution.** Distributions of average stride length for each participant in 6MWT. Orange distribution represents the average stride length as calculated by the reference standards. Blue distribution represents the average stride length as calculated by the CMPedometer algorithms. **a)** Shows absolute frequency vs. average patient stride length and **b)** shows relative frequency vs. average patient stride length.
